# The Role of Sirtuin-1 in Immune Response and Systemic Lupus Erythematosus

**DOI:** 10.3389/fimmu.2021.632383

**Published:** 2021-04-26

**Authors:** Yueqi Qiu, Xingyu Zhou, Yu Liu, Siqi Tan, Yaping Li

**Affiliations:** Department of Dermatology, Second Xiangya Hospital, Central South University, Changsha, China

**Keywords:** systemic lupus erythematosus, Sirtuin-1, pathogenesis, immune cells, epigenetics, histone deacetylase

## Abstract

Systemic lupus erythematosus (SLE) is a potentially fatal multisystem inflammatory chronic disorder, the etiology and pathogenesis of which remain unclear. The loss of immune tolerance in SLE patients contributes to the production of autoantibodies that attack multiple organs and tissues, such as the skin, joints, and kidneys. Immune cells play important roles in the occurrence and progression of SLE through amplified immune responses. Sirtuin-1 (SIRT1), an NAD^+^-dependent histone deacetylase, has been shown to be a pivotal regulator in various physiological processes, including cell differentiation, apoptosis, metabolism, aging, and immune responses, via modulation of different signaling pathways, such as the nuclear factor κ-light-chain-enhancer of activated B cells and activator protein 1 pathways. Recent studies have provided evidence that SIRT1 could be a regulatory element in the immune system, whose altered functions are likely relevant to SLE development. This review aims to illustrate the functions of SIRT1 in different types of immune cells and the potential roles of SIRT1 in the SLE pathogenesis and its therapeutic perspectives.

## Introduction

Systemic lupus erythematosus (SLE), a typical prototype of connective tissue diseases, is characterized by immunocomplex-mediated inflammation. SLE is caused by an inappropriate immune response to nucleic acid containing cellular particles from both the innate and adaptive immune systems. The complexity of SLE is indicated by involvement of multiple organs and various clinical features, such as arthritis, nephritis, vasculitis, and pleuritis or pericarditis, and abnormal laboratory examinations, such as decreased complements and increased levels of autoantibodies including antinuclear antibodies (ANAs), anti-Smith (Sm) antibodies, and anti-double-stranded DNA (dsDNA) antibodies (1). Although the pathogenesis of SLE has been explored from the level of histopathology to molecular biology, the pathogenic mechanisms underlying this disease remain unclear. In recent years, a growing number of studies have pointed out the important roles of epigenetic modifications in the progression of autoimmune disorders (2). Epigenetic modifications can be described as modulation of gene expression without changing the original sequence of the genetic code or inheritable and reversible alterations during transcription activity (3). Epigenetic mechanisms in SLE mainly include DNA methylation, histone modification, and non-coding RNA modification, among which DNA hypomethylation was the first epigenetic pattern determined in SLE patients (4).

Histone deacetylases (HDACs) can regulate protein stability and function mainly in three ways: by directly deacetylating histones H3, H4, and H1; by promoting modifications in the methylation of histones or DNA; or by interacting and deacetylating transcription factors, coregulators, and (or) DNA repair proteins (5). Human HDACs can be categorized into four classes. Sirtuin-1 (SIRT1), a class III HDAC, is distinguished from classes I, II, and IV HDACs by its NAD^+^-dependency. SIRT1 participates in a wide range of vital activities such as cell differentiation, apoptosis, metabolism, aging, and immune response. It is also involved in the production and differentiation of both innate and acquired immune cells, which have been illustrated in many immune-mediated disease models. SIRT1 overexpression was found in CD4^+^ T cells of a murine lupus model, suggesting that upregulated SIRT1 may contribute to the SLE pathogenesis (6). However, SIRT1-null mice presented with immunoglobulin deposition in the kidneys as well as a high level of serum anti-nuclear antibody, which resembled the symptoms of lupus nephritis (7). Moreover, resveratrol (RSV), an activator of SIRT1, has been shown to be protective against pristane-induced lupus mice, when alleviation of proteinuria and decreased deposition of immune globulin in the kidneys were observed (8). Therefore, the expression and activity of SIRT1 in the occurrence and progression of SLE are elusive and complicated. In this review, we provide some insights into the current understanding of the functions of SIRT1 in the SLE pathogenesis and its therapeutic perspectives for SLE.

## The Structure and Enzymatic Mechanism of SIRT1

**Figure 1:**
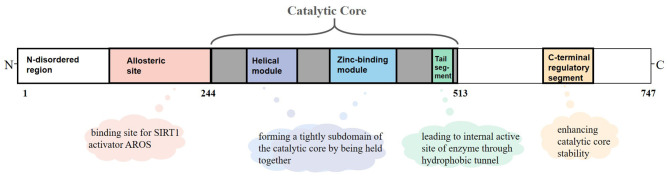
Overview of sirtuin-1 (SIRT1) structure. SIRT1 is a monomer with 747 amino acids, which consists of a well-structured catalytic core (244–513 residues), the N-terminal (1–243 residues), and the C-terminal (513–747 residues) regions. In the catalytic core domain, the helical module and the Zn^2+^ binding module are connected via an extensive hydrophobic interface. The tail segment imitates the p53 peptide sequence, binding to the internal activation site of enzyme through the hydrophobic tunnel. The allosteric site is considered as a binding site for active regulator of SIRT1 (AROS). The C-terminal regulatory (CTR) segment in disordered regions of C-terminal has a profound impact on the stability of catalytic domain.

SIRT1 is an NAD^+^-dependent protein present predominantly in a monomer form and composed of 747 amino acids, which consists of three primary structuring elements: a well-structured catalytic core domain ranging between 244 and 512 residues and two highly disordered terminal regions comprising N-terminal (1–243 residues) and C-terminal (513–747 residues) regions. The catalytic core where NAD^+^-dependent deacetylation reactions occur is a highly conservative structure and consists of two subdomains. The Rossman fold domain is one of the subdomains, which is characterized by a large hydrophilic domain equipped with proteins that bind NAD^+^/NADH. The other subdomain is a small one comprising a helical module and a Zn^2+^ binding module. These two modules connect with each other via an extensive hydrophobic interface (9). Between the large and small subdomains, there is a catalytic pocket that has been categorized into three different sites (A, B, and C) according to the positions of adenine, ribose, and nicotinamide of NAD^+^ (10). Although the N-terminal and C-terminal regions are chaotic, they are critical for catalysis because segments having important functions exist in these regions (11). RSV and other hydrophobic sirtuin-activating compounds activate SIRT1 enzyme by mimicking active regulator of SIRT1 (AROS), which binds to an allosteric site in the N-terminal region. Further, a specific structure named the C-terminal regulatory (CTR) segment, which exists in the disordered regions of the C-terminal portion and interacts with the catalytic core, has a profound impact on the stability of the catalytic domain (12). In addition, the tail segment of the catalytic domain binds CTR either in the presence of a substrate or in an inactive condition. This segment imitates the p53 peptide sequence, binding to the internal activation site of the enzyme through the hydrophobic tunnel, which might be of great value to develop competitive SIRT1 inhibitors on the basis of this substrate-mimicking interaction (9) (Figure 1).

SIRT1 deacetylation is NAD^+^ dependent, although this feature is not usually observed in amidohydrolases. SIRT1 catalyzes deacetylation by transferring the acetyl group from acetyllysine in the protein to NAD^+^ (13). Some reports indicate that the sequential kinetic mechanism of SIRT1-catalyzed deacetylation begins with a ternary complex composed of SIRT1, the acetylated substrate, and NAD^+^. After ternary complex formation, nicotinamide cleavage from NAD^+^ occurs first, followed by release of the deacetylated product and then the novel metabolite O-acetyl-ADP-ribose (OAADPR) (14). Catalysis is regulated by the NAD^+^ concentration or the NAD^+^/NADH ratio. Nicotinamide phosphoribosyl transferase (NAMPT), the rate-limiting enzyme in NAD^+^ synthesis, is responsible for maintaining SIRT1 enzymatic activity under different pathophysiological conditions (15), while nicotinamide (NAM) reduces SIRT1 activity through a negative feedback loop. Pyrazinamidase/nictinamidase 1 (PNC1) deaminates NAM to niacin through a remedial pathway to reduce the accumulation of NAM (16). OAADPR is also an important metabolic byproduct and acts as a signaling molecule for gating of the transient receptor potential melastatin-related channel 2 (TRPM2) (17, 18). OAADPR may display gene silencing by facilitating the assembly and loading of the Sir2–4 silencing complex onto nucleosomes (16).

## Post-Translational Modification of SIRT1

Redox and metabolic states regulate the activity of SIRT1 depending on the NAD^+^/NADH ratio. A variety of chemical modifications of SIRT1 are associated with the pathophysiology of different diseases.

### Phosphorylation

Phosphorylation is a common protein post-translational modification in which the side chains of amino acids (such as serine, threonine, and tyrosine) in the protein are esterified with the phosphate group, thereby altering the conformation and activity of the protein. Nasrin et al. found that activation of c-Jun N-terminal kinase 1 (JNK1) induced by reactive oxygen species (ROS) results in the direct phosphorylation of Ser47 in human SIRT1, promoting the nuclear aggregation of SIRT1. Subsequently, deacetylation of histone H3 by SIRT1 is enhanced (19). Cyclin-B/Cdk1 is a cell cycle-dependent kinase that is activated at the G2/M cell cycle checkpoint and inactivated at the late stage of cell proliferation. Protein profiling showed that SIRT1 Thr530 and Ser540 are substrates of Cdk1. When Thr530 and Ser540 are phosphorylated by Cdk1, SIRT1 activity is upregulated. In addition, non-phosphorylated SIRT1 is in an inactive state (20). The dual-specificity tyrosine-phosphorylated and -regulated kinase (DYRK) is a family of highly conserved protein kinases that autophosphorylate their own tyrosine residues, as well as serine and threonine residues of exogenous substrates. Protein immunoprecipitation and GST pull-down assays confirmed that DYRK1A and DYRK3 could interact with SIRT1 and phosphorylate Thr522 of SIRT1, promoting deacetylation of p53 (21). AMP-activated protein kinase (AMPK) is a serine/threonine kinase composed of a catalytic α subunit and regulatory β and γ subunits. AMPK interacts with the deacetylase activity domain of SIRT1, *in vivo* and *in vitro*, and directly phosphorylates SIRT1 at Thr344, releasing SIRT1 from its endogenous inhibitor, leading to an increase in deacetylated p53 levels (22). The Janus kinase (JAK)-signal transducer and activator of transcription (STAT) pathway is a critical signaling mechanism for many cytokines and growth factors (23). JAK1 was found to phosphorylate SIRT1 at Tyr280 and Tyr301, both of which are highly conserved and located in the SIRT1 catalytic domain. However, instead of directly altering the SIRT1 deacetylase activity, JAK1-mediated phosphorylation intensifies SIRT1-mediated suppression of downstream STAT3 acetylation and transcriptional activity (24). In general, the deacetylation of SIRT1 is impeded by reduced SIRT1 activity after phosphorylation.

### Other Modifications

S-glutathionylation is also an important post-translational modification, in which cysteine residues of proteins interact with glutathione (GSH) to form disulfide bonds. Using mass spectrometry, several cysteine residues of SIRT1 have been shown to be modified by S-nitrosoglutathione (GSNO), including Cys67, which is S-glutathiolated; although this change had no effect on basal SIRT1 activity, it weakened the activation by SIRT1 activator (25). Small ubiquitin-related modifier (SUMO) is a member of the ubiquitin protein family. Under normal physiological conditions, Lys734 of human SIRT1 is modified by SUMO to maintain transcriptional silencing and genomic stability. Mutation of SIRT1 at Lys734 or deSUMOylation by SUMO-specific protease 1 (SENP1), a nuclear deSUMOylase, significantly reduced SIRT1 deacetylase activity, resulting in an increase in the levels of acetylated p53 (26, 27). Inflammation increases the abundance of inducible nitric oxide synthase (iNOS), resulting in high levels of nitric oxide (NO), which can mediate the regulation of a range of proteins by S-nitrosylation. Shinozaki et al. showed that the ability of SIRT1 to bind zinc is disrupted by NO-induced S-nitrosylation. Furthermore, inhibition of SIRT1 deacetylation and activation of p53 and p65 acetylation lead to apoptosis and proinflammatory responses (28).

## Activators and Inhibitors of SIRT1

### Activators of SIRT1

RSV (trans-3,4,5-trihydroxystilbene), a dietary polyphenol often found in foods such as grapes and red wine, is the earliest molecule reported to be a SIRT1 activator and has many health benefits, including improved metabolism, reduced inflammation, protective endothelial cell function, and prevention of tumorigenesis (29). Much evidence suggests that RSV indirectly increases the expression of SIRT1 by upregulating levels of NAD^+^ through activation of AMPK. Elevated NAD^+^ levels subsequently contribute to higher deacetylation of SIRT1 substrates (30). More recently, a study revealed that RSV interacts with a specific N-terminal domain and a 7-amino-4-methylcoumarin (AMC)-containing peptide, which is also responsible for the regulation of SIRT1 activation (31). Although high absorption (70%) of RSV via oral ingestion was shown, systemic bioavailability of RSV is almost only 0.5% due to hepatic or intestinal glucuronidation and sulfation (32). Because of the RSV's poor bioavailability, a commercial micronized RSV formulation named SRT501 was widely used, which mimics the beneficial effects of caloric restriction like RSV (33). SRT1720, a SIRT1 activator with a different structure from RSV, binds and activates the enzyme at the amino acids 183–225 N-terminal to the core domain, which is same to RSV. This compound could stimulate 750% SIRT1 activity at 10 μM (34). There are also some activators unrelated to RSV. Isoflavones are involved in mitochondrial biogenesis through PGC-1α (peroxisome proliferator-activated receptor-γ coactivator 1-α) activation and increase SIRT1 activity and/or expression in renal proximal tubular cells at a concentration of 10 μM (35). Dao et al. isolated four terpenylated coumarins from a tree called *Ailanthus altissima*, which was found through the SIRT1 activator screening program. They then confirmed that these four compounds enhanced SIRT1 activity at a dose of 10 μM by a SIRT1-NAD/NADH assay *in vitro* and a SIRT1-p53 luciferase assay *in vivo* (36). Some molecules from traditional Chinese medicine have SIRT1-activating property, including ginsenoside Rb2, ginsenoside Rc, and schisandrin A (37). Furthermore, with the development of bioinformation and chemosynthesis technology, a number of synthetic SIRT1 activators have appeared, such as pyrroloquinoxalines, dihydropyridines, imidazothiazoles, oxazolopyridines, and related analogs (9).

### Inhibitors of SIRT1

The activity of SIRT1 is regulated by various internal or exogenous substances. As each deacetylation of SIRT1 requires one NAD^+^ molecule, the modulation of SIRT1 activity is closely related to the availability of NAD^+^. Nicotinamide, a well-known endogenous inhibitor of SIRT1, is a reaction product of NAD^+^, which non-competitively inhibited SIRT1 with IC50 < 50 μM by negative feedback regulation of NAD^+^. Similarly, NAD^+^-glycosylated hydrolases downregulate the expression of SIRT1 by hydrolyzing NAD^+^. Besides, some endogenous factors such as miR-34a and tumor necrosis factor-α (TNF-α) inactivate SIRT1 (38, 39). In a large-scale fluorimetric screening research, indole derivatives that are potential SIRT1 inhibitors have been found with IC50 values ranging from 60 to 100 nM (40). Other exogenous SIRT1 inhibitors such as EX-527, tetrahydrocarbazole, thiobarbiturates, thiocyanates, and phloroglucinol derivatives have also been intensively studied (9, 41).

## The Immunoregulatory Roles of SIRT1 in Immune Cells

**Figure 2:**
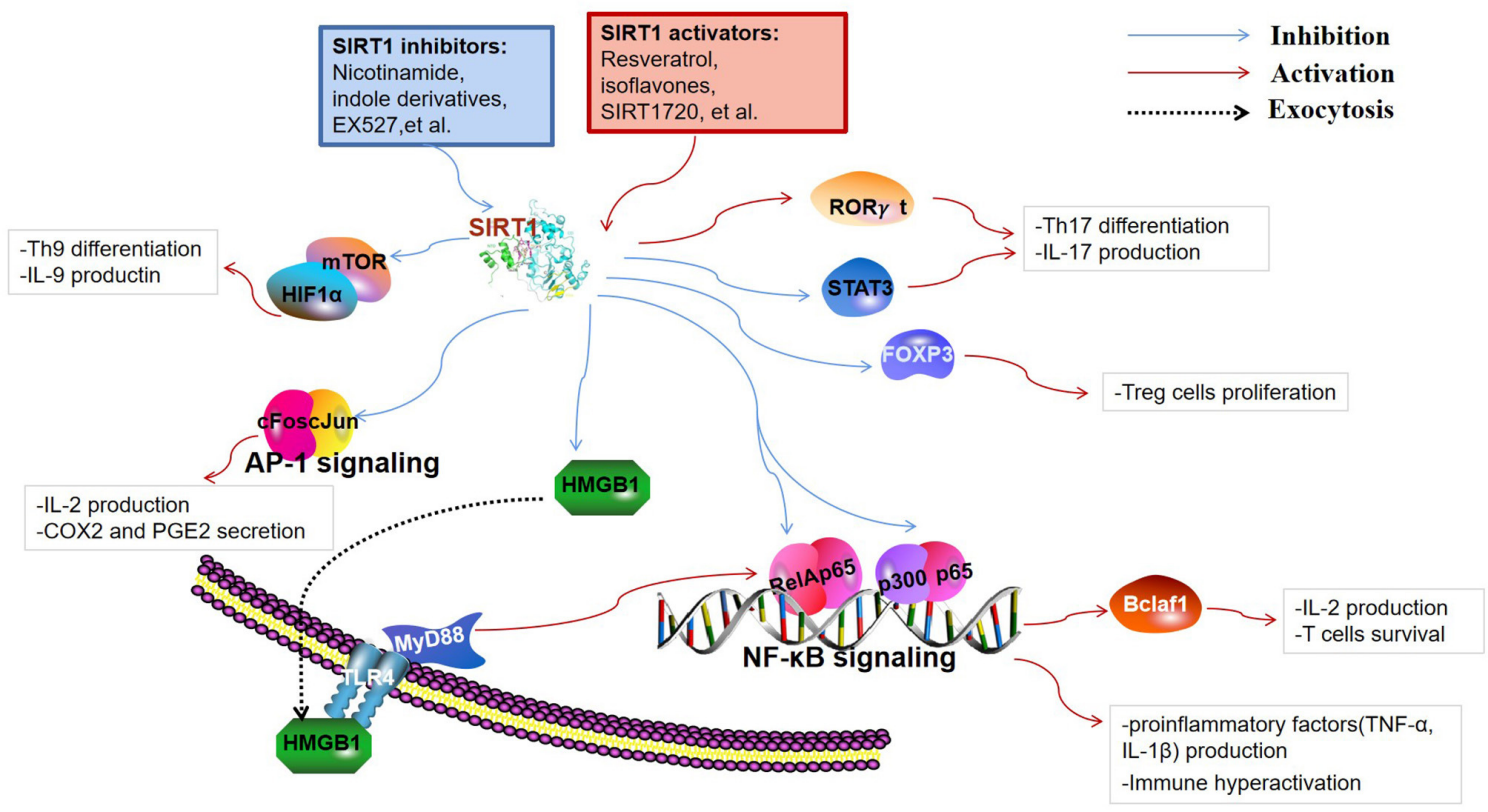
Targets of SIRT1 in the immune responses. SIRT1 inhibits immune activity via deacetylation of multiple targets. Nuclear factor κ-light-chain-enhancer of activated B cells (NF-κB) and activator protein 1 (AP-1) are key transcription factors involved in the production of inflammatory cytokines and immune hyperactivation. B-cell lymphoma 2-associated factor 1 (Bclaf1) was found to be regulated by NF-κB, which is associated with immune cell survival. SIRT1 restrains the high-mobility group box 1 (HMGB1) exocytosis to exert protective and anti-inflammatory effects by inhibiting the HMGB1/TLR4/MyD88/NF-κB signaling pathway. Fork head box P3 (FOXP3), signal transducer and activator of transcription 3 (STAT3), and retinoid acid receptor-related orphan receptor gamma t (RORγt) are pivotal regulators of T cell differentiation into regulatory T cells (Tregs) and Th17 cells. Th9 cell differentiation and the release of IL-9 are regulated through SIRT1-mTOR-HIF1α signaling.

SIRT1 are involved in a broad range of fields, including cellular apoptosis and proliferation, calorie restriction, metabolism, inflammation, immune function, tumorigenesis, and autophagy. Thus far, most studies have indicated that decreased SIRT1 expression or activity contributes to the enhancement of lymphocyte activation, thereby leading to the occurrence of autoimmune disease. The immunoregulatory function of SIRT1 is closely related to the cell types and the specific substrates that are targeted to the immune response. Besides, SIRT1-mediated regulation of metabolic processes is also critical for immune cell function. This section mainly introduces the immunoregulatory role and detailed targets of SIRT1 in immune cells (Figure 2).

### SIRT1 in Innate Immune Cells

Macrophages are the most vital and irreplaceable components of the innate immune system and are the primary source of inflammatory cytokines such as TNF-α, interleukin (IL)-1, IL-6, and so on. They are functionally polarized into M1 (classical) and M2 (alternative) macrophages under various microenvironmental stimuli. M1 macrophages, polarized by interferon (IFN)-γ, play a critical role against viral and bacterial infections, whereas M2 macrophages, stimulated by IL-4 and IL-13, are closely related to responses against inflammatory reactions, fibrosis, and tumorigenesis (42). Deletion of SIRT1 increased the expression of M1 molecule iNOS, but suppressed the expression of M2 molecules, including arginase 1 (Arg1) and mannose receptor (MR), in a macrophage SIRT1 knockout mouse model. Although macrophage SIRT1 participated in the regulation of M1/M2 polarization, the exact regulatory mechanism of SIRT1 transcription is unknown (43). Pharmacological SIRT1 inhibition has been shown to negatively regulate the G1/S transition and cell cycle progression by inhibition of the transcriptional activation of E2F1 and Myc gene, indicating that SIRT1 inactivation restrains macrophage proliferative capacity during bone marrow-derived differentiation (44). The knockdown of SIRT1 in the mouse macrophages broadly activate the JNK and IKK inflammatory pathways and increases TNF-α secretion (45). Nuclear factor κ-light-chain-enhancer of activated B cells (NF-κB) is a constitutively expressed transcription factor that controls the expression of many genes involved in inflammation. SIRT1 can reduce the transcription activity of NF-κB dimers by deacetylating RelA/p65 at the lysine 310 residue. It further inhibits the production of downstream cytokines including TNF-α and IL-1β in macrophages (46). In addition to directly deacetylating p65, SIRT1 possibly inhibits the transcription of p65 target genes in combination with p65 and p300 to interfere with the NF-κB pathway. Recent studies have shown that SIRT1 activation can reduce the secretion of high-mobility group box 1 (HMGB1) and moderate the subsequent HMGB1/TLR4/MyD88/NF-κB signaling pathway to exert protective and anti-inflammatory effects against neurological disorders (47, 48). Qian et al. demonstrated that increased SIRT1 activity inhibited the expression of the TLR2 gene and was associated with CD38 deficiency-mediated inflammation in macrophages through SIRT1/NF-κB/TLR2 signaling (49). Beyond the NF-kB pathway in macrophages, SIRT1 also played a major part in activator protein 1 (AP-1) signal of the immune response. SIRT1 directly binds to c-Fos and c-Jun domains, thereby inhibiting the AP-1 activity. Deacetylation of c-Jun diminishes inflammation resulting from the decreased expression of cyclooxygenase 2 (COX2) and the reduced production of prostaglandin E2 (PGE2) (50). Myeloid-derived suppressor cells (MDSCs) are immune cells that belong to a heterogeneous set of myeloid cells, which suppress immune response in autoimmunity, cancer, and other pathological conditions to maintain immune cell tolerance (51). Hypoxia-inducible transcription factor-1 alpha (HIF1α) subunit, an indispensable factor in cellular metabolism, was also involved in SIRT1-associated immune responses. The activity and differentiation of MDSCs were limited by SIRT1 through HIF1α-dependent glycolysis (52). Neutrophilic granulopoiesis is highly dependent on granulocyte colony-stimulating factor (G-CSF). SIRT1 activation upregulates the granulocyte-specific transcription factors and then leads to the enhanced expression of G-CSF and its receptor through the NAMPT/NAD^+^/SIRT1 pathway, resulting in positive feedback management of G-CSF (53). Dendritic cells (DCs) are specialized antigen-presenting cells and an initiator of the adaptive immune responses. For instance, DC-derived IL-12 can support the development of T helper 1 (Th1) cells, whereas transforming growth factor (TGF)-β1 can promote the production of regulatory T cells (Tregs). SIRT1 deficiency in DCs upregulated levels of IL-12 and decreased the expression of TGF-β1 through HIF1α-dependent pathway. Therefore, blockade of SIRT1 in DCs restrained Tregs generation while promoting Th1 cell differentiation and development, leading to an intensive T cell-mediated inflammation (54). The absence of SIRT1 in DCs could lead to inappropriate metabolic processes through the increased expression of acetyl-CoA carboxylase (ACC1), which enhances mitochondrial dysfunction and induces pathologic innate immune cytokines (55).

### SIRT1 in Adaptive Immune Cell Responses

CD4^+^ helper T cells play a pivotal role in directing the appropriate responses for host immune defense in several immune-mediated inflammatory diseases (56). Tregs inhibit undesirable immune responses against self-antigens or bacteria, and their reduced number and/or function have been found in several autoimmune diseases (57). Human Tregs are characterized by the factor fork head box P3 (FOXP3), CD25 (IL-2 receptor α), and CD127 (IL-7 receptor α) (58). Levels of FOXP3 protein are critical for a well-balanced immune system. It is clear that in addition to gene transcription and translation, post-translational modifications regulate the expression and/or activity of SIRT1 (59). There are three lysine acetylation sites (K31, K262, and K267) in murine FOXP3 or SIRT1-mediated deacetylation (60). In addition to the post-translational control of FOXP3, in some studies, genetic deletion or pharmacologic inhibition of SIRT1 improved the amount and function of FOXP3^+^ Tregs by increasing FOXP3 mRNA level as well (60, 61).

Th17 has inflammatory properties and induces the expressions of IL-17A, IL-21, and IL-22 as well as TNF-α (62). Some disease models indicated that differentiation of Th17 cells was disrupted by the degree of deacetylation of STAT3. SIRT1 activator impairs Th17 development and does not inhibit SIRT1 (39, 63, 64). However, SIRT1 has a proinflammatory role in Th17 cell generation and function and enhances deacetylated RORγt (retinoid acid receptor-related orphan receptor gamma t) transcriptional ability. Deacetylated RORγt has stronger transcriptional activity than FOXP3, thus strengthening Th17 phenotype. Therefore, SIRT1 activation induces IL-17 production by increasing RORγt stability, which maintains the balance between Th17 and Tregs (65).

Th1 cells secrete cytokines including TNF-α, IFN-γ, IL-2, and granulocyte-macrophage colony-stimulating factor (GM-CSF) and Th2 cytokines including IL-4, IL-5, and IL-6 (66, 67). The upregulated expression of SIRT1 due to RSV treatment impeded the activation of CD4^+^ T cells and secretion of IFN-γ, indicating that the activated SIRT1 negatively impacted Th1 differentiation and IFN-γ production (68). B-cell lymphoma 2-associated factor 1 (Bclaf1), primarily considered as a promoter of cellular apoptosis, proved to be critical for T cell activation (69). Kong et al. reported that SIRT1 suppresses Bclaf1 activity by inhibiting NF-κB transcriptional signaling as well as by deacetylating histone lysine residues at the Bclaf1 promoter region, resulting in the decreased level of IL-2 and apoptosis of T cells (70). HIF1α activity is always associated with the generation of proinflammatory cytokines and restriction of anti-inflammatory cytokines (71). SIRT1 deficiency promotes Th9 cell differentiation and the release of IL-9 via the SIRT1-mTOR-HIF1α signaling-coupled glycolytic pathway, which is possibly involved in antineoplastic immune and chronic allergic airway inflammation (72). However, some studies have indicated that SIRT1 could be a proinflammatory protein. SIRT1 is a target of microRNA-142 that downregulates SIRT1 expression and attenuates the migration capacity of CD4^+^ T cells (73, 74). In a mouse model of ovarian cancer, Th1 cell differentiation of CD4^+^ T cells was induced after the treatment of artesunate, a promoter of microRNA-142 expression leading to the reduced expression of SIRT1 (68), indirectly suggesting a positive role for SIRT1 in Th1 differentiation. Given the above information, SIRT1 has multi-faceted regulatory roles in CD4^+^ T cell differentiation and inflammatory cytokines secretion.

CD8^+^ T cell activation is important for protective immune response against viral and bacterial infections. Basic leucine zipper transcription factor, ATF-like (BATF), provoked by IL-12 and required for CD8^+^ cell survival, promotes CD8^+^ T cell differentiation by inhibiting SIRT1 (75). The transcription factor forkhead box protein O1 (FOXO1) is involved in the transcriptional reprogramming of CD8^+^ T cells and has been identified as a SIRT1 target. At a reduced SIRT1 level, FOXO1 was found to be degraded; enhanced glycolytic and cytotoxic capacities of CD8^+^ T cells were also noted, resulting in immune dysfunction (76).

Although the exact function of SIRT1 in B cells has not been well-illustrated, there have been some studies revealing that SIRT1 cooperates with CD38 and NAD^+^ to regulate B cell maturation (77). SIRT1 level is suppressed by the overexpression of miR-132, which leads to exaggerated lymphotoxin and TNF-α production in normal B cells (78). In recipients transplanted with SIRT1^−/−^ T cells, splenic B cell reconstitution was increased, which reduced the differentiation of plasma cells (79). Activation-induced cytidine deaminase (AID) is a critical molecule for maturation of antibody response. SIRT1 deletion or downregulation in B cells inhibited deacetylation of Aicda promoter histones, DNMT1, and NF-κB p65, while induced AID expression, resulting in enhanced T-independent antibody responses. This effect can be reversed by SRT1720 (80). Therefore, SIRT1 deficiency may restrain the differentiation and activation of B cells into plasma cells, enhance pro-inflammatory cytokine secretion from B cells, and promote the generation of autoantibodies, which could be a possible reason for autoimmune disorders and explain the occurrence of symptoms similar to lupus nephritis in SIRT1-null mice in addition to the failure of autophagy (7) (Table 1).

**Table 1:** The function of SIRT1 in immune cells.

**Cell type**	**Subjects**	**Intervention**	**Activity of SIRT1 (native)**	**Targets**	**Effects**	**References**
Macrophages	C57BL/6 mice	Knockout of SIRT1	Deletion	Unknown	M1 macrophages induce iNOS ↑ M2 molecules including Arg1 ↓and MR ↓	(43)
	Maf-DKO macrophages	NAM (0–10 mM)	Suppressed	E2F1, Myc, FOXOs	Cell cycle progression ↓	(44)
	RAW264.7 cells	RSV(50 μM)	Elevated	JNK and IKK	The expression of TNF-α ↓	(45)
	RAW264.7 cells	RSV (20 μM)	Elevated	NF-κB p65	Expression of TLR2 ↓	(49)
	RAW264.7 cells HEK293 cells	Transfected with AdSIRT1 RNAi	Suppressed	c-Fos, c-Jun (AP-1)	COX2↓and PGE2 ↓	(50)
MDSCs	C57BL/6 mice	Knockout of SIRT1	Deletion	mTOR-HIF1α	Glycolytic activity in splenic MDSC ↓ Directs M1-MDSC differentiation	(52)
Neutrophils	CD34^+^ bone marrow progenitor cells from healthy individuals	The substrate of NAMPT (10 ng/ml)	Elevated	C/EBP-β	Expression of G-CSF ↑ and G-CSF receptor ↑	(53)
Dendritic cells	Normal human DCs (CC-2701; Lonza)	EX-527 (10 μM)	Suppressed	HIF1α	IL-12 ↑ and expression of TGF-β1 ↓	(54)
	C57BL/6J mice	Knockout of SIRT1	Deletion	AMPK-ACC1	Mitochondrial dysfunction ↑ Pathologic innate immune cytokines ↑	(55)
CD4^+^ T cells	293T, Jurkat cells GFP-Foxp3 knock in mice (C57BL/6J)	Ex-527 (40 mg/kg *in vivo* and 50 μM *in vitro*)	Suppressed	FOXP3	Differentiation and stability of Tregs ↑	(60)
	C57BL/6 mice	Methylene blue (20 mg/kg)	Elevated	AMPK/SIRT1/NF-κB	Th17 cell generation ↑	(64)
	Human CD4^+^ T cells from healthy volunteers	RSV, SRT720 (10 μM)	Elevated	STAT3	Th17 cell differentiation ↓	(63)
	C57BL/6	Ex-527 (10 mg/kg)	Suppressed	RORγt (K69, K81, and K99)	IL-17 ↓ Th17 cell development ↓	(65)
	Sirt1^−/−^ mice	Knockout of SIRT1	Deletion	Bclaf1 (H3K56 residues at CNS3 and CNS4)	IL-2 ↑ T cell apoptosis ↑	(70)
	C57BL/6 mice	Knockout of SIRT1	Deletion	mTOR-HIF1α	Th9 cell differentiation ↑ IL-9 ↑	(72)
	C57BL/6 mice	Artesunate (0/10/50/100 mg/kg)	Suppressed	miR-142/SIRT1	IFN-γ ↓ Th1 differentiation ↓ Cell apoptosis ↑	(68)
CD8^+^ T cells	C57BL/6 mice	Knockout of BATF	Suppressed	BATF/SIRT1/c-Jun	CD8^+^ T cell differentiation ↑	(75)
	Human CD8^+^ T cells	RSV (25/50 μM)	Elevated	FOXO1	Glycolytic and cytotoxic capacities of CD8^+^ T cells ↓	(76)
B cells	Mouse B lymphocytes BaF3	Transfected with SIRT1 vector or shRNA	Elevated	NF-κB	Plasma cell differentiation ↓ Pro-inflammatory cytokines secretion ↑ The expression of p53 ↓	(81)
	C57BL/6 mice	Knockout of SIRT1	Deletion	Aicda promoter histones; NF-κB p65; DNMT1	AID expression ↑ Generation of autoantibodies ↑	(80)

*iNOS, inducible nitric oxide synthase; Arg1, arginase 1; MR, mannose receptor; NAM, nicotinamide; FOXOs, factor forkhead box protein Os; RSV, resveratrol; NK, natural killer cell; COX2, cyclooxygenase 2; PGE2, prostaglandin E2; NAMPT, nicotinamide phosphoribosyl transferase; G-CSF, granulocyte colony-stimulating factor; TGF-β, transforming growth factor-β; AMPK, AMP-activated protein kinase; FOXP3, factor fork head box P3; RORγt, retinoid acid receptor-related orphan receptor gamma t; Bclaf1, B-cell lymphoma 2-associated factor 1; BATF, basic leucine zipper transcription factor; DNMT1, DNA methyltransferase 1, AID, activation-induced cytidine deaminase*.

## The Potential Roles and Therapeutic Prospects of SIRT1 in SLE

### SIRT1 in Immune Cells and SLE

The occurrence and development of SLE is associated with malfunction in both the innate and adaptive immune system, resulting in damage to immunological tolerance and autoantibody production. T cells and their cytokines are critical factors in SLE pathogenicity. IL-17A, IL-22, and IL-23, cytokines of Th17, recruit neutrophils, facilitate inflammatory cell tissue infiltration, and promote antibody production and inflammation in SLE. However, few research articles about SIRT1-mediated mechanisms in SLE associated with innate immune cells were published. This part revolves around the correlation between SIRT1 and adaptive immune system of SLE.

Aryl hydrocarbon receptor (AhR) is a transcription factor involved in various inflammatory diseases, whose ligand is necessary for the differentiation and maturation of CD4^+^ T cells into Th17 or Tregs. The peripheral blood of patients in the active stages of SLE manifested a noteworthy AhR activation (90). SIRT1 activation has been shown to reverse an AhR-induced imbalance between the populations of Th17 and Tregs and the upregulation of IL-17A and IL-22 levels in CD4^+^ T cells (91, 92). Later studies in lupus CD4^+^ T cells demonstrated that ultraviolet B strongly activates AhR, which subsequently contributes to the low expression of SIRT1 by binding to the SIRT1 promoter. As a result, reduced SIRT1 levels resulted in the inhibition of DNA methyltransferase 1 (DNMT1) in CD4^+^ T cells from patients with SLE (82). IL-2, another key T cell-derived cytokine, regulates FOXP3 expression in Tregs to maintain immune tolerance. In adoptive experiments using murine SLE models, the transfer of Tregs has proven to prevent disease progression, including improved renal pathology and reduced mortality of mice with lupus (57). The development and maturity of Tregs highly rely on IL-2 levels, and lower levels of IL-2 in every differentiated subset of CD4^+^ T cells were discovered in SLE patients than in those without SLE (93). As mentioned above, in T cells, FOXP3 could be directly deacylated by SIRT1 to suppress Tregs proliferation. SIRT1 suppresses IL-2 transcription by regulating Bclaf1 and nuclear factor of activated T cells (46). Th17 numbers are always positively related to disease activity and severity, because a higher proportion of Th17 cells and serum IL-17 levels are found in SLE patients with active symptoms compared to healthy controls (94). SIRT1 serves as a regulator of restraining Th17 differentiation to maintain the balance between Tregs and Th17 cells, which might be crucially involved in the SLE pathogenesis. However, the direct evidences whether SIRT1 participates in the SLE pathogenesis by interfering with IL-2 synthesis and the balance between Tregs and Th17 cells have not yet been illustrated. According to a study investigating the protective effects of RSV in an animal model of lupus nephritis, the ratio of Th1 to Th2 cells and the number of Th1 cells decreased after RSV treatment and showed correlation with the dosage (8). Recently, human umbilical cord-derived mesenchymal stem cell (hUC-MSC) transplantation has been proved to be effective to treat SLE, while the underlying mechanisms are not clear. MiR-199a-5p was identified as one of the key players to promote senescence of splenic CD4^+^ T cells and alleviate lupus-associated symptoms in MRL/lpr mice, through the downstream SIRT1/p53 pathway (83). Beyond CD4^+^ cells, SIRT1 inhibition weakens CD8^+^ T cell cytotoxicity in SLE patients, thereby being impressionable to COVID-19, according to a review based on a search conducted in Medline, Scopus, and WOS (95). Also, COVID-19 poses an increased risk to SLE patients (96). Katsuyama et al. present evidence that CD38 expression is elevated in CD8^+^ T cells in SLE patients with high incidence of infections. CD8CD38^high^ T cells are capable to decrease T cell cytotoxic responses by suppressing the expression of cytotoxic-related transcriptional factors through an NAD^+^/SIRT1/EZH2 axis that could be reversed by EZH2 inhibitors (84).

SLE was originally thought to be a disease mediated by abnormal B cells and plasma cells, because systemic inflammation was instigated by excessive production of autoantibodies from B cells (97). B cell hyperactivity and impaired regulation of B cells lead to not only the generation of autoantibodies but also enhancement of the ability of antigen presentation to T cells (98, 99). Based on the complicated mechanism of B cells in the occurrence of SLE, mouse B lymphocyte BaF3 cell line was used to investigate the potential function of SIRT1 in SLE pathogenesis. It was found that the overexpression of SIRT1 substantially improved BaF3 cell vigor and viability, prevented apoptosis, and increased cytokine production by regulating the NF-κB pathway (81). The proinflammatory function of the overexpression of SIRT1 in BaF3 is slightly different the from protective role of that in B cells extracted from C57BL/6 mice (80). In MRL-lpr mice, it was found that RSV caused a remarkable reduction in B cells, especially plasma cells, in the spleen and bone marrow, resulting in decreased levels of serum autoantibodies such as IgG1 and IgGα. This is because RSV enhances the expression of Fcγ receptor (FcγRIIB), a receptor for IgG, which leads to repressed B cell receptor-mediated activation and induces B cell apoptosis (85). Furthermore, plasma cells with high expression of FcγRIIB were remarkably decreased in response to RSV treatment, causing a decrease in immunocomplex deposition in the kidney (100). This is clinically significant in SLE therapy because plasma cells generated from the bone marrow cannot be efficiently eliminated by either anti-proliferative agents or anti-CD20 mAbs (85). As neither T cells nor NK cells express FcγRIIB, the use of FcγRIIB as a relatively specific target of humoral immunity avoided the disruption of cell-mediated immune balance, minimized the side effect of immunosuppression, and is considered an excellent therapeutic strategy for SLE (101, 102).

Although RSV was unable to abrogate autoantibody formation (86), the traditional Chinese prescription Lang Chuang Fang (LCF), which can also increase the expression of SIIRT1, was shown to downgrade dsDNA autoantibody and improve lupus nephritis (87). Another Chinese herbal medicine named Honokiol (HNK) was capable of alleviating manifestation of the severe form of lupus nephritis (AFLN) by negative regulation of T cell functions and by enhancement of SIRT1/autophagy axis activation (101, 102). In Consiglio's study, SIRT1 promoter rs3758391 was implicated to modify SLE morbidity, while rs3758391 T allele may contribute to a higher systemic lupus erythematosus disease activity index (SLEDAI) and lupus nephritis (103).

### SIRT1 in Apoptotic Processes and SLE

Enhanced apoptosis and deficiencies in the clearance of apoptotic cells contribute to the presentation of nuclear autoantigens, inducing autoantibodies, and driving aberrant immune responses in SLE (104). Chromatin condensation and DNA fragmentation are major characteristics of apoptosis. Post-translational modifications of histones can affect the structure and function of chromatin during apoptosis (105, 106). HDAC inhibitors can reset histone hypoacetylation, resulting in improved clinical manifestation; however, high concentrations of HDAC inhibitors may induce apoptosis (106, 107). This dual character of HDAC inhibitors suggests that the involvement of SIRT1 in apoptosis is potentially implicated in SLE etiopathogenesis. SIRT1-mediated regulation of p53 deacetylation prevents apoptosis, and when SIRT1 interacts with FOXO1 or FOXO3, cell cycle arrest is increased and cell death is avoidable (108–111). SIRT1 can reverse miR-181a-mediated oxidative stress-induced FOXO1 acetylation and apoptosis *in vitro* and *in vivo* (112). Taurine-upregulated gene 1 (TUG1), an lncRNA that can regulate miR-223 and SIRT1 expression, showed a protective function against inflammatory injury in a lipopolysaccharide (LPS)-induced LN cell model by repressing apoptosis and reducing inflammatory factor secretion through the modulation of PI3K/AKT and NF-κB signaling pathways (89). SIRT1 overexpression can inhibit apoptosis and promote BaF3 cell proliferation and pro-inflammatory cytokine release by modulating the NF-κB pathway, implying that SIRT1 might be a dangerous factor in SLE (81) (Table 2). It is well-known that anti-dsDNA antibodies fluctuate with SLE activity and are closely related to manifestations of severe lupus (113, 114). Although the exact mechanism of the generation of anti-dsDNA antibodies remains unclear, previous studies have shown that, in SLE, extracellular DNA can also be produced upon cell death (115–117). Olivares et al. found that serum anti-dsDNA antibody levels were significantly associated with urinary SIRT1 mRNA levels in LN patients, which indicates that SIRT1 affects the process of apoptosis. However, an abnormal state of apoptosis is not the only characteristic of SLE; the observed increase in urinary SIRT1 mRNA levels may only be a response to an active inflammatory state (118).

**Table 2:** The functional studies of SIRT1 in SLE.

**Study type**	**Subjects**	**Intervention**	**Pathways**	**Effects**	**References**
*In vitro*	CD4^+^ T cells from diagnosed active SLE patients	Transfection with SIRT1 siRNA; SRT1720 (10/20 μM)	AhR-SIRT1-DNMT1	Suppressed SIRT1 expression caused by UVB activating AhR inhibited DNMT1 activity in CD4^+^ T cells of SLE patient expressions.	(82)
*In vivo*; *In vitro*	BALB/c mice; CD4^+^ T cells and CD19^+^ B cells from splenic mononuclear cells	Fed with RSV (50/75 mg/kg/day); RSV (0, 10, 20, 40, or 80 μM)	Not mentioned	RSV inhibits IgG levels in pristane-induced lupus mice, B cell proliferation, and antibody production *in vivo*; RSV induces CD4^+^ T cell apoptosis and the percentage of Th1 cells and decreases the ratio of Th1/Th2 cells *in vitro*.	(8)
*In vivo*; *In vitro*	MRL/lpr and ICR mice; CD4^+^ T cells from mononuclear spleen suspensions	Transfused with 5 × 10^5^ hUC-MSCs via the tail vein; EX527 (50 μM), SRT1720 (5 μM)	MiR-199a-5p /SIRT1/p53	HUC-MSCs decreased SIRT1 expression and increased senescence of splenic CD4^+^ T cells both *in vivo* and *in vitro*.	(83)
*In vitro*	CD8 T cells from SLE patients; Jurkat cell line	EX527 GSK126 (EZH2 inhibitor)	CD38/NAD^+^/SIRT1/EZH2	CD38 increased acetylated EZH2 through inhibition of SIRT1, leading to decreased cytotoxic capacity of CD8 T cells.	(84)
*In vitro*	Mouse B lymphocytes BaF3	Transfected with SIRT1 vector or shRNA against SIRT1	SIRT1/NF-κB	Overexpression of SIRT1 promoted BaF3 cell viability, inhibited apoptosis, and upregulated pro-inflammatory cytokines.	(81)
*In vivo*	MRL/lpr mice	Intra-peritoneal injections of RSV (20 mg/kg/q2d)	SIRT1/FcγRIIB/NF-κB	RSV upregulated the expression of FcγRIIB on B cells and myeloid cells, resulting in ameliorating lupus nephritis and disease activity.	(85)
*In vivo*	Pristane-induced SLE murine model	Fed with RSV (25/50 mg/kg/day)	Not mentioned	Low dose of RSV increased IFN-α and IL-6 and mitigated TNF-α. High dose of RSV decreased creatinine levels and proteinuria. RSV is not proved to inhibit autoantibody production.	(86)
*In vivo*	MRL/lpr mice	Orally with LCF granule (0.97, 1.9, and 3.90 g/kg/day)	Not mentioned	LCF granule upregulated the expression of SIRT1 and Nrf2 and p65 NF-κB was reduced. Levels of proteinuria, BUN, and SCr decreased in LCF granule-treated mice.	(87)
*In vivo*; *In vitro*	NZB/W F1 mice	Fed with HNK (30 mg/kg)	SIRT1/autophagy axis/NLRP3	HNK improved renal function and albuminuria and reduced IgG anti-dsDNA production in ASLN mice by enhancing the SIRT1/autophagy axis inhibition of the NLRP3 inflammasome.	(88)
*In vitro*	HK-2 cell line	Transfected with pEX-TUG1 or sh-TUG; pEX-SIRT1 (or sh-SIRT1) transfection	UTG1/miR-223/SIRT1/PI3K/AKT; NF-κB	TUG1 prevented lipopolysaccharide (LPS)-simulated LN cell from inflammatory injury by regulating miR-223 and SIRT1 expression.	(89)

*AhR, aryl hydrocarbon receptor; DNMT1, DNA methyltransferase 1; hUC-MSCs, human umbilical cord-derived mesenchymal stem cells; LCF, Lang Chuang Fang; BUN, blood urea nitrogen; SCr, serum creatinine; TUG1, taurine-upregulated gene 1*.

## Discussion

SIRT1, an NAD^+^-dependent monomer protein, possesses anti-inflammatory functions and regulates immune cell differentiation primarily by inhibiting the transcriptional activity of pro-inflammatory factors through deacetylation. The exact pathogenetic mechanism underlying SLE is still not understood due to various internal and/or external pathogenic factors participating in the intricate process of disease development. FOXP3, RORγt, STAT3, RelA/p65 (NF-κB), and c-Fos/c-Jun (AP-1) are transcription factors targeted by SIRT1, which are well-studied and are crucial to a balanced immune system, but the role of SIRT1 in autoimmune disease is only in the incipient stage, especially in the progression of SLE. The relationship between T cells and SIRT1 has been paid more attention to compared to B cells, so research is needed to illustrate the function of SIRT1 in various types of cells in the immune system. AhR and FcγRIIB could be SIRT1 therapeutic targets in SLE to avoid side effects of anti-inflammatory and steroids synthetic cortisone medications and to solve the problem that current treatments do not lead to improved curative effect for severe patients. SIRT1 is always seen as an anti-inflammatory factor. For example, RSV alleviates inflammation and decreases the level of autoimmune antibodies. However, SIRT1 overexpression has been found in CD4^+^ T cells from MRL/lpr mice model. Urinary mRNA SIRT1 levels depend on the degree of disease activity; however, it is unknown whether the high levels of SIRT1 result from or result in the progression of SLE. There is lack of clinical evidence proving the efficacy of SIRT1 activator in humans. Besides, RSV encounter difficulties in application to humans due to its poor water solubility, poor bioavailability, and poor dose. Beyond RSV, more types of pharmacological SIRT1 inhibition or activation should be applied to the SLE model. SIRT1 activators were widely studied *in vivo*, but few reports mentioned whether side effects or adverse events occurred. The functions of SIRT1 have been explored in various autoimmune diseases, and SIRT1 has shown its polymorphism in immune regulation and keeping SIRT1 expression in the appropriately balanced status (instead of over- or under-expression), which may be necessary for abnormally functioning immune system. However, studies about the direct relationship between SIRT1 expression and SLE pathogenesis are still in an original state, implying that there is still a long way to solve this problem.

## Author Contributions

YQ and YaL made substantial contributions to the conception and design and the acquisition of data. All authors have participated in the analysis and interpretation of data and drafting the article or revising it critically for important intellectual content. All authors gave final approval of the version to be submitted and any revised version.

## Conflict of Interest

The authors declare that the research was conducted in the absence of any commercial or financial relationships that could be construed as a potential conflict of interest.
